# Editorial: Improving responses to immunotherapy in glioblastoma multiforme

**DOI:** 10.3389/fimmu.2024.1407930

**Published:** 2024-04-23

**Authors:** Stephanie E. B. McArdle, Divya Nagarajan, Michael E. Barish

**Affiliations:** ^1^ John van Geest Cancer Research Centre, Nottingham Trent University, Nottingham, United Kingdom; ^2^ School of Science and Technology, Department of Biosciences, Nottingham Trent University, Nottingham, United Kingdom; ^3^ Pencil Biosciences Limited, Macclesfield, United Kingdom; ^4^ Department of Stem Cell Biology and Regenerative Medicine, Beckman Research Institute, City of Hope, Duarte, CA, United States

**Keywords:** glioblastoma multiforme (GBM), immunotherapy, responses, improve, future

Glioblastoma multiforme (GBM), WHO grade 4 glioma, IDH-wildtype, is essentially a uniformly fatal primary brain tumour. Whilst many therapeutic interventions have been studied pre-clinically, and tantalizing observations have emerged from clinical trials, very few have matured to the level required for clinical use as therapies for patients with GBM, and unfortunately nearly all GBM tumours relapse.

These tumours are uniquely challenging due to their location within the brain, making delivery of therapeutic interventions difficult. Further, the GBM tumour microenvironment is highly immunosuppressive, dominated by tumour-associated macrophages (TAMs) and myeloid suppressor cells. Here, Takacs et al., who previously reported the existence of three populations of myeloid cells within the glioma microenvironment based on expression of chemokine receptors CCR2 and CX3CR1, demonstrate that myeloid-derived suppressor cells (MDSCs) expressing both CCL2 and CCL7 represent a potent and migratory T cell suppressive population, and that a therapeutic strategy targeting CCR2 might help limit recruitment of this population to the brain. Wei et al. discuss therapy-induced changes of the tumor microenvironment (TME) in recurrent GBM (rGBM), with large infiltration of CD68+ macrophages following anti-angiogenic therapy together with the almost (82%) complete loss of the immunogenetic epidermal growth factor receptor variant III (EGFRvIII). These macrophages are the dominant non-malignant cells in the TME of rGBM, are far more diverse than a simple binary M1/M2 polarisation and are very plastic cells that need to be taken into consideration when applying immunotherapies to treat rGBM as reviewed by Wei et al. This immunosuppressive microenvironment also affects tumour-infiltrating lymphocytes (TILs), which have been found to exhibit exhausted phenotypes, expressing PD-1, TIM-3, LAG-3, TIGIT, and CD39, or are otherwise dysfunctional. Zhao et al. describe 25 immune cell types in 796 GBM samples, find patterns associated with different clinical outcomes, and identify novel dysregulated signalling pathways that could be used as prognosticators of treatment outcomes. In this regard, Gutova et al. focus on one such pathway, the Wnt signalling pathway, using the small molecule inhibitor ICG-001, and find that it has pleotropic effects on a GL261 tumour model: increased TIL recruitment and activation, modulation of the tumour stroma, and differentiation of self-renewing glioma stem cells. However, as demonstrated by lorgulescu et al., murine GL261 and CT2A glioma models, in contrast to human GBM, have high mutational loads, and neither cell line shares the essential genetic or histologic features of human GBM. As such, these results warrant confirmation using better suited GBM models.

Adoptive transfer of chimeric antigen receptor (CAR) T cells are a potentially interesting avenue. Karachi et al. discuss this approach in their review, considering ongoing clinical trials and the hurdles faced by CAR T cell strategies. One challenge is antigen loss in the context of highly heterogeneous tumors, rendering single antigen-targeted T cells useless. Indeed, antigen escape has been demonstrated, and may be one of several mechanisms underlying tumour recurrence. One could envisage adoptive transfer of CAR T cells recognising more than one antigen, or CAR T cells recognizing targets expressed by most or all cells within a tumour, or strategies incorporating additional CAR T cells to be injected once the tumour recurs. Relevant to this approach, Rose et al. identify multiple surface proteins, including some that are mutated proteins, some that are targeted by existing drugs, and some novel proteins not yet targeted.

In this Research Topic, two groups, Kang et al. and Nabors et al., discuss γδ T cells as vehicles for CAR expression and as a potential alternative to the more abundant αβ T cells. γδ T cells are a small (0.5-5%) subset of all T cells, whose T cell receptors (TCRs) consist of γ and δ chains, hence their name. Contrary to αβ TCRs, antigen recognition by γδ TCRs is independent of class I major histocompatibility complex (MHC) molecules, and therefore potentially obviate the very expensive requirement for patient-specific autologous adoptive transfer. Further, γδ T cells produce high numbers of cytokines and are the most abundant T cell in the gut mucosa. Nabors et al. previously showed that GBM cells constitutively express low levels of the stress associated NKG2D ligands (NKG2DL) recognised by γδ T cells. They show here that NKG2DL expression is increased by temozolomide (TMZ) treatment, but also that TMZ is toxic to γδ T cells. However, by rendering their γδ T cells resistant to TMZ, these cells could be administrated to patients receiving TMZ treatment in a first-in-human phase 1 clinical trial (NCT04165941). CAR engineered γδ T cells could both be an alternative approach to CAR T cell immunotherapy, and a complementary approach to be considered on tumour recurrence.

One hopes that the efforts described here, and others, will collectively begin to help patients with GBM that presently lack effective treatment options.

## Author contributions

SM: Writing – original draft, Writing – review & editing. DN: Writing – review & editing. MB: Writing – review & editing.

